# Quantitative Benefit–Risk Assessment of Vaccination Against COVID‐19: A Systematic Review

**DOI:** 10.1002/pds.70099

**Published:** 2025-01-29

**Authors:** E. Claire Newbern, Lea Wildisen, Rita Verstraeten, Corinne Willame, Kevin Haynes, Bennett Levitan, Nicolas Praet

**Affiliations:** ^1^ Johnson & Johnson Innovative Medicine, Global Epidemiology Horsham Pennsylvania USA; ^2^ Johnson & Johnson Innovative Medicine Basel Switzerland; ^3^ Johnson & Johnson Innovative Medicine Beerse Belgium; ^4^ Johnson & Johnson Innovative Medicine Titusville New Jersey USA

**Keywords:** COVID‐19, quantitative benefit–risk assessment, vaccination

## Abstract

**Purpose:**

With the introduction of COVID‐19 vaccines, there has been a proliferation of quantitative benefit‐risk assessments (qBRAs). Prior work on other types of vaccines has found that published qBRAs have not always clearly reported methods and/or results needed to assist in the application of the qBRA findings. The aim was to systematically identify, review, and critically assess published COVID‐19 vaccine qBRA. The ultimate goal is to support the future development of robust qBRA for existing, new, and updated vaccines.

**Methods:**

We systematically reviewed COVID‐19 vaccine qBRAs identified from multiple sources through April 17, 2023, including literature databases, selected Health Authority websites, and a grey literature search. We critically assessed whether key features typical of qBRA were presented in these reports.

**Results:**

We identified 37 COVID‐19 vaccine qBRAs from screening 2220 publications and 18 other sources. The qBRAs were conducted on two mRNA and two adenoviral vector COVID‐19 vaccines. Only one qBRA represented low‐ and middle‐income countries. Although many qBRAs used simple calculations (*n* = 25), more complex models were presented in 15 reports. Simple approaches were able to employ stratification by age and/or sex to highlight safety issues affecting specific demographic groups and scenarios to account for changes in viral transmission and vaccine effectiveness over time. Details regarding data sources and analytic methods were missing or limited in some reports.

**Conclusions:**

This comprehensive description and critical assessment of COVID‐19 vaccine qBRAs together with available guidance can be used to support the development of robust and transparent future vaccine qBRAs.

**Summary**

- This systematic review catalogues recent quantitative benefit–risk assessments of COVID‐19 vaccines.
- This review highlights issues with information presented in COVID‐19 vaccine quantitative benefit–risk assessments, such as data sources, methods, results, and interpretation of findings.
- This review supports the need for further guidance regarding the conduct and reporting of quantitative benefit–risk assessments.

## 1 Introduction

Weighing benefits and risks of vaccination is the basis for informed public health decision‐making on immunization recommendation and vaccine use. While qualitative evidence is often relied upon to assess the benefit–risk profile of vaccines, quantitative approaches are increasingly used [1]. The field of medicinal product benefit–risk assessment (BRA) has indeed advanced considerably over the last two decades, including the application of structured BRA frameworks and quantitative benefit–risk assessment (qBRA). Structured benefit–risk evaluation aims at providing an objective assessment of the benefit–risk profile of medicinal products and a higher transparency for decision‐making purposes [2, 3]. In practice, a qBRA consists of comparing the number of averted health outcomes due to the targeted disease with the number of adverse reactions caused by the vaccine [1, 2, 3, 4, 5, 6, 7]. In other words, qBRA integrates numerical estimates of benefits and risks, such as counts or rates, along with clinical judgment of the clinical impact or weight of each benefit and risk, offering a more robust foundation to support decision‐making.

A recent systematic literature review identified vaccine qBRA publications and described approaches and methods used by researchers conducting these qBRAs [1]. They found that the majority of reports published prior to the COVID‐19 pandemic were conducted in high‐income countries and targeted the rotavirus, dengue, and influenza vaccines. They also highlighted heterogeneity in the methods used and the approaches taken to describe methodologies and results.

With millions of deaths and hundreds of millions of cases, COVID‐19 is responsible for a high burden on public health that led to saturation of health care systems, especially during epidemic peaks. The spectrum of SARS‐CoV‐2 infection severity ranges from asymptomatic or mild symptomatic infections to events requiring hospitalization or resulting in death. Epidemiological data collected since the start of the pandemic indicate that COVID‐19 transmission and severity have evolved over time and have varied in impact regionally [8]. To address the high medical need associated with COVID‐19, several vaccines were rapidly developed and introduced as one of the prevention measures against the disease. In this accelerated environment, rapid generation of evidence on safety and effectiveness of introduced vaccines was critical to guide immunization recommendations. This evidence included assessment of benefit–risk profiles of vaccines.

Since the launch of COVID‐19 vaccination at the end of 2020, there has been a proliferation of COVID‐19 vaccine qBRAs, which have been made available publicly in the published literature, Health Authority reports or proceedings from National Immunization Technical Advisory Group (NITAG) COVID‐19 vaccine recommendation meetings. The aim of this paper was to systematically identify, review and critically assess COVID‐19 vaccine qBRA methods, supporting parameters and results. Because benefit–risk assessments are often conducted for Health Authority decision‐making, we took a broad approach to identify qBRAs. Our ultimate goal was to support researchers in developing robust qBRA in the future for existing, new, and updated vaccines.

## 2 Methods

### 2.1 Search Strategies and Report Selection

We used multiple strategies to identify COVID‐19 vaccine qBRAs: (1) a systematic literature review, (2) a targeted review of prespecified Health Authority websites, (3) a general internet search for grey literature, and (4) a review of reference lists from selected qBRA reports. Identified reports were managed using Rayyan, a free online reference management tool [9]. A report had to meet the following criteria to be included in the review: (1) assess COVID‐19 vaccine(s), (2) include quantitative (absolute or relative) measures of benefits and risks of the assessed vaccine(s), (3) be written in English, and (4) not present qBRA results included in another reviewed report.

The systematic literature review followed the standard Preferred Reporting Items for Systematic Review and Meta‐Analyses (PRISMA) guideline [10]. Searches of PubMed, ScienceDirect, and medRxiv covered January 1, 2020, to April 17, 2023 (last date of database exploration). The search strategy applied both of the following sets of terms to identify publications: (1) SARS‐CoV‐2 vaccine and (2) benefit–risk (Supporting Information 1). Articles identified in literature databases were first de‐duplicated and then two epidemiologists independently screened the de‐duplicated reports in a two‐step process (LW and RV). In the first step, titles and abstracts were screened. In the second step, the full text of reports retained in the first step was assessed. Discrepancies were settled through consensus‐based discussion between the two reviewers.

For the other sources of potential qBRA, we reviewed Health Authority websites for the United States, European Union (EU), Australia, Japan, and the World Health Organization (WHO) and used defined search terms (Table S1) in a general internet search. The last date of search for these sources was May 30, 2023. Reports from these sources were excluded when their results were already included from the literature database search (*n* = 3) or were not considered qBRA (*n* = 1).

### 2.2 Data Extraction

Information was extracted from identified qBRAs with consideration of items presented in a previously published vaccine qBRA literature review [1]. Extracted data included (1) basic study details (authors, publication date/type, geographic location, target population, and study period), (2) characteristics of vaccine(s) considered (brand(s); primary or booster regimen; comparison groups (e.g., no vaccination or comparison to another vaccine); and reported vaccine effectiveness/efficacy measured used with targeted outcome and duration of protection), (3) the analytic approach (benefit and risk outcomes, data sources and measures, analytic techniques (e.g., simple deterministic calculation, statistical probabilistic analysis, complex modeling techniques), benefit–risk measure, stratifications, and sensitivity analysis including scenario analysis (scenarios refer to combinations of assumptions that provide a sensitivity analysis to baseline assumptions)), and (4) reported conclusions on the benefit–risk profile. A Microsoft Excel spreadsheet with headings corresponding to the above study elements was used to extract information from identified reports. Three epidemiologists (LW, RV, and ECN) extracted this information. If the information for a specific study element could not be identified in a report, then this was noted as “not identified” in the spreadsheet. The specified elements of the reports were tabulated and summaries for key elements were included in a figure.

## 3 Results

**FIGURE 1:**
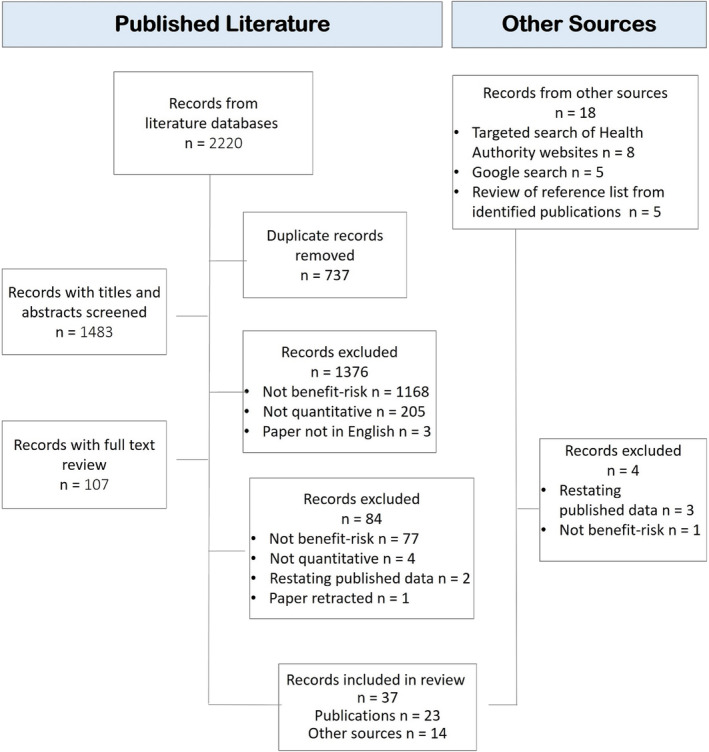
Record review and selection process.

We identified 2220 articles in literature databases (Figure 1). After removing duplicates (*n* = 737), the review by epidemiologists excluded articles primarily for not being considered a benefit–risk assessment (*n* = 1245) or a quantitative benefit–risk assessment (*n* = 209). In total, 37 records met the inclusion criteria, 23 from the publication databases, and 14 from other sources.

**FIGURE 2:**
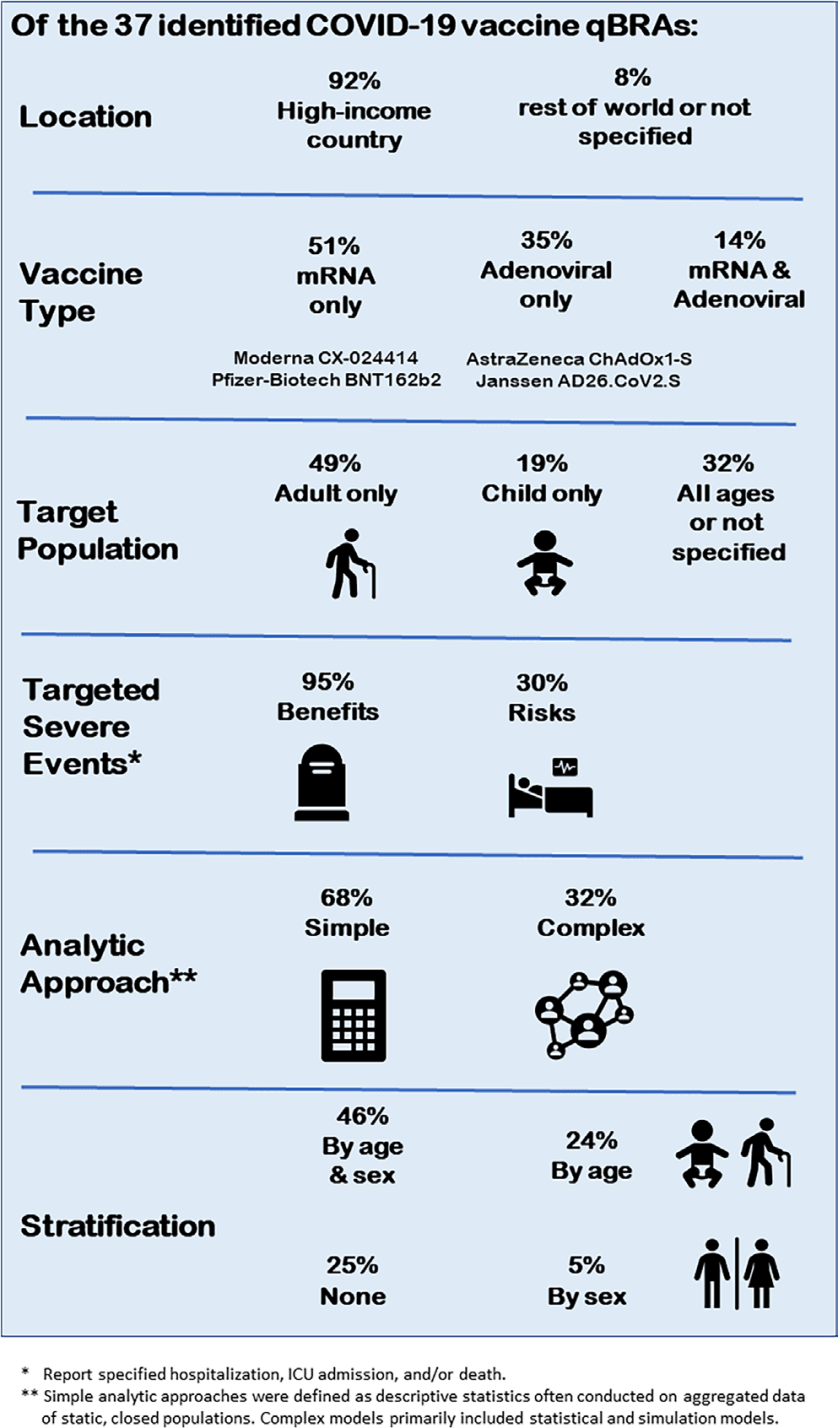
Characteristics of identified COVID‐19 vaccine quantitative benefit–risk assessments (qBRAs) (*N* = 37).

Selected characteristics of the identified COVID‐19 vaccine qBRAs are summarized in Table 1 and Figure 2. Health authorities conducted the majority of identified qBRAs (*n* = 21), 12 were conducted solely by academic researchers, two involved a sponsor company, and two were performed by authors with other affiliations. The earliest COVID‐19 vaccine qBRAs identified were available in April 2021 with similar numbers of reports produced in 2021 (*n* = 17) and 2022 (*n* = 19). Identified qBRAs included published articles (*n* = 21), published health authority reports from public health updates (e.g., MMWR or Eurosurveillance) (*n* = 6), and reports or presentations posted on websites (*n* = 10).

**TABLE 1:** Characteristics of identified quantitative benefit–risk assessments (*n* = 37).

First author, affiliation, publication year [Reference #]	Income group (region/country) target population	Vaccine(s) regimen comparator (e.g., no vaccine)	Study period	Vaccine effectiveness outcome duration	Benefit measures data sources	Risk measures data sources	Analytic model type	Stratified, scenario, sensitivity analysis	Benefit–risk assessment measures	Benefit–risk balance conclusions reported
Australian government Health Authority 2021 [11]	HIC (Australia) Adults	AstraZeneca Primary No vaccine	4 m before June 2021	Not reported COVID‐19 severe outcomes 4 m	Hospitalizations, ICU admissions, and deaths Not stated	TTS National pharmacovigilance	Simple calculation (deterministic)	Age Transmission	Rate	No interpretation provided in report
Bardosh, K. Academic 2022 [12]	HIC (US) Adults	mRNA Booster Primary series	Omicron	Not reported COVID‐19 cases and severe events 6 m	Hospitalizations National disease surveillance	Myocarditis/pericarditis and SAEs Clinical trials	Simple calculation (deterministic)	Age and sex N/A VE/durability	Rate	Does not favor vaccine
Corrao, G. BMC ID Academic/Health Authority 2022 [13]	HIC (EU/Italy) Children—Adults	mRNA Primary No vaccine	December 2020–November 2021	Measured COVID‐19 severe outcomes 28 d	Hospitalizations, ICU admissions, and deaths Generated in study	Myocarditis/pericarditis hospitalization Generated in study	Statistical analysis (probabilistic)	Age and sex	Number needed to treat/harm	Favors vaccine
Corrao, G. vaccines Academic/Health Authority 2022 [14]	HIC (EU/Italy) Children—Adults	Multiple: AstraZeneca, Moderna, Pfizer Primary No vaccine	May 2021	55%–89% COVID‐19 hospitalizations and deaths Median = 35 d	Hospitalizations, ICU admissions, and deaths Generated in study	Thrombosis Generated in study	Statistical analysis (probabilistic)	Age and sex N/A VE/durability	Number needed to treat/harm	Favors vaccine
Devriese, S Health Authority 2021 [15]	HIC (EU/Belgium) Adults	Janssen Primary No vaccine	September 2020–March 2021	85% COVID‐19 severe outcomes 4 m	Hospitalizations, ICU admissions, and deaths Regional disease surveillance	TTS Regional pharmacovigilance	Simple calculation (deterministic)	Age Transmission VE/durability	Rate	No interpretation provided in report
EMA 2021 [16]	HIC & MIC (EU) Adults	AstraZeneca Primary No vaccine	Through April 2021	60% and 80% COVID‐19 cases and hospitalizations Not reported	Hospitalizations, ICU admissions, and deaths Regional disease surveillance	TTS Regional pharmacovigilance	Simple calculation (deterministic)	Age Transmission	Rate	Favors vaccine
Funk, PR. Health Authority 2022 [17]	HIC (US) Children—Adults	Pfizer Primary No vaccine	December 2020–July 2021	70%–90% and 8090%–90% COVID‐19 cases and hospitalization 6 m	Cases, hospitalizations, ICU admissions, and deaths National disease surveillance	Myocarditis/pericarditis cases, hospitalizations, and deaths Optum and Vaccine Safety Datalink	Simple calculation (deterministic)	Age and sex N/A VE/durability	Rate	Favors vaccine
Gurdasani, D. Academic 2021 [18]	HIC (UK/England) Children	mRNA Primary No vaccine	September–December 2021	Actual measured COVID‐19 cases and severe outcomes 4 m	Cases, hospitalizations, ICU admissions, deaths, and Long COVID National disease surveillance	Myocarditis Publications	Simple calculation (deterministic)	Sex Transmission	Rate	Favors vaccine
Hawkes, MT. Academic 2022 [19]	HIC (Australia and Canada) Children	Pfizer Primary No vaccine	12 m before February 2022	27%, 86%, and 96.7% COVID‐19 infection, hospitalization, and death Not reported	Cases, hospitalizations, ICU admissions, deaths, and MIS‐C Publications	Anaphylaxis, myocarditis Publications	Complex model Dynamic (simulation, probabilistic)	Age Transmission	Benefit–Risk ratio	Favors vaccine
JCVI Health Authority 2022 [20]	HIC (UK) Children	Pfizer Primary No vaccine	Omicron	Not reported COVID‐19 cases Not reported	Hospitalizations, ICU admissions, deaths, and MIS‐C Publications	Myocarditis UK and VAERS pharmacovigilance	Simple calculation (deterministic)	N/A	Rate	Favors vaccine depending on age
Kalafat, E. Academic 2022 [21]	N/A Adults	mRNA Primary No vaccine	Not reported	Not reported COVID‐19 infections and severe outcomes Not reported	ICU admissions, deaths, and pregnancy/birth outcomes Publications	Myocarditis and pregnancy outcomes Publications	Simple calculation (deterministic)	N/A	Rate	Favors vaccine
Krug, A. Academic 2022 [22]	HIC (US) Children	Pfizer Primary No vaccine	Separately for Delta and Omicron	81.0%–93.0% (Delta) and 44%–64% (Omicron) COVID‐19 hospitalization Not reported	Hospitalizations Publications	Myocarditis/pericarditis VAERS	Simple calculation (deterministic)	Age and sex	Rate	Does not favor vaccine
Lau, CL. Academic 2021 [23]	HIC (Australia) Children—Adults	AstraZeneca Primary No vaccine	August–September 2021	Multiple by variant (Alpha and Delta) COVID‐19 cases and deaths Not reported	Deaths National disease surveillance	TTS deaths National pharmacovigilance	Complex model Bayesian network (deterministic)	Age and sex Transmission	Rate	Favors vaccine depending on age
Lewis, G. Academic 2022 [24]	N/A N/A	mRNA Primary No vaccine	N/A	Distributions of VE COVID‐19 cases and death Not reported	Infections and deaths WHO/Publications	Severe Adverse Events (SAE) Clinical trials	Complex model Dynamic (simulation, probabilistic)	N/A Transmission VE	Surface of equipoise	Favors vaccine for high‐risk individuals
Liu, Y. Academic/Health Authority 2022 [25]	MIC (Europe/Albania, Armenia, Azerbaijan, Belarus, Bosnia—Herzegovina, Bulgaria, Georgia, Moldova, Russia, Serbia, North Macedonia, Turkey, and Ukraine) Adults	AstraZeneca Primary Alternative vaccination strategies	March 2021–December 2022	Multiple COVID‐19 transmission, infections, severe outcomes, and death 3 m	Severe outcomes Publications	TTS deaths Publications	Complex model Dynamic (simulation, probabilistic)	Age Transmission VE/durability	Benefit–risk ratio	No interpretation provided in report
MacIntyre, CR. Academic/Health Authority 2021 [26]	HIC (Australia) Adults	AstraZeneca Primary No vaccine	12 m through June 2021	100% COVID‐19 deaths Not reported Other scenario considered	Deaths National disease surveillance	TTS deaths National pharmacovigilance	Simple calculation (deterministic)	N/A Transmission	Rate	Favors one vaccine over another
MacNeil, JR. Health Authority 2021 [27]	HIC (US) Adults	Janssen Primary No vaccine	Through April 2021	65% and 90% (Janssen) and 85% and 95% (mRNA) COVID‐19 infection and severe outcomes 6 m	Hospitalizations, ICU admissions, and deaths National disease surveillance	TTS VAERS	Simple calculation (deterministic) and Complex model dynamic (simulation)	Age and sex Transmission VE/durability	Rate	Favors vaccine
Mammen, M. MAH 2021 [28]	HIC (US) Adults	Janssen Primary No vaccine	Through March 2021	100% COVID‐19 hospitalizations and deaths Not reported	Hospitalizations and deaths National disease surveillance	TTS‐like cases MAH pharmacovigilance	Simple calculation (deterministic)	Age and sex	Rate	Favors vaccine
Mayfield, HJ. Academic 2022 [29]	HIC (Australia) Children—Adults	AstraZeneca Primary No vaccine	6 m before April 2022	Not reported COVID‐19 cases and deaths Not reported	Cases, deaths, and blood clots National disease surveillance	TTS cases and deaths National pharmacovigilance	Complex model Bayesian network (deterministic)	Age and sex Transmission	Rate	No interpretation provided in report
Moon, J Other 2021 [30]	HIC (US) N/A	mRNA Primary No vaccine	March–September 2021	Not reported COVID‐19 cases Not reported	Hospitalizations, ICU admissions, and deaths National disease surveillance	Adverse Events (AE) VAERS	Simple calculation—Clavien‐Dindo GRADE (deterministic)	N/A	Benefit–risk ratio	No interpretation provided in report
Myers, V. Academic 2022 [31]	HIC (Israel) Children—Adults	Pfizer Primary No vaccine	Dec 2020—November 2021	N/A	Cases and severe outcomes National disease surveillance	Myocarditis National pharmacovigilance	Simple calculation (deterministic)	Age and sex	Rate	Favors vaccine
Oliver, S. ACIP Health Authority 2021 [32]	HIC (US) Adults	Janssen Primary No vaccine	Through November 2021	69%–76% COVID‐19 hospitalization 6 m	Hospitalizations National disease surveillance	GBS, Myocarditis, TTS cases and deaths VAERS	Simple calculation (deterministic)	Age and sex N/A VE/durability	Rate	Favors one vaccine over another
Oliver, SE. MMWR Health Authority 2022 [33]	HIC (US) Adults	Multiple: Janssen, mRNA Primary No vaccine	Through November 2021	Not reported COVID‐19 hospitalization 6 m	Hospitalizations National disease surveillance	GBS, Myocarditis, TTS hospitalizations and deaths ACIP	Simple calculation (deterministic)	Age and sex N/A VE/durability	Rate	Favors one vaccine over another
Ouldali, N. Academic/Health Authority 2022 [34]	HIC (EU/France) Children	mRNA Primary No vaccine	N/A	N/A	Hyperinflammatory syndromes National disease surveillance	Hyperinflammatory events including MIS‐C National pharmacovigilance	Simple calculation (deterministic)	Sex	Rate	Favors vaccine
Palladino, R Academic. 2021 [35]	HIC (EU/Italy) Adults	AstraZeneca Primary No vaccine	Nov 2020–April 2021	72% Not reported 8 m	Deaths Publications	Thrombotic events Publications	Complex model Monte Carlo (simulation, probabilistic)	Age N/A VE/durability	Benefit–Risk ratio	Favors vaccine depending on age
Rosenblum, HC. MMWR Health Authority 2021 [36]	HIC (US) Adults	Multiple: Janssen, mRNA Primary No vaccine	February–July 2021	90% (Janssen) and 95% (mRNA) COVID‐19 severe outcomes 6 m	Cases, hospitalizations, ICU admissions, and deaths National disease surveillance	GBS, myocarditis, TTS VAERS	Simple calculation (deterministic) and Complex model dynamic (simulation)	Age and sex N/A VE/durability	Rate	Favors vaccine
Rosenblum, H. ACIP Health Authority 2021 [37]	HIC (US) Children—Adults	Multiple: Pfizer, Janssen Primary No vaccine	Through July 2021	74.6% and 84% COVID‐19 cases and hospitalization 6 m	Cases, hospitalizations, ICU admissions, and deaths National disease surveillance	Myocarditis VAERS and Vaccine Safety Datalink	Simple calculation (deterministic)	Age and sex N/A VE/durability	Rate	Favors vaccine
Shiri, T. Academic/MAH 2022 [38]	HIC (UK) Children—Adults	mRNA Primary & Booster No vaccine	3 m before December 2021	88% and 91% COVID‐19 hospitalizations 3 m	Cases, hospitalizations, ICU admissions, and deaths	Myocarditis/pericarditis VAERS	Complex model Dynamic (simulation, probabilistic)	Age Transmission VE/durability	Rate	Favors vaccine
Sinclair, JE. Academic 2022 [39]	HIC (Australia) Children—Adults	Pfizer Primary and Booster No vaccine	2 m before August 2022	Multiple by age COVID‐19 cases and deaths Multiple (< 2, 2–3, 4–5 m)	Cases and deaths National disease surveillance	Myocarditis cases and deaths National pharmacovigilance and VAERS	Complex model Bayesian network (deterministic)	Age and sex Transmission VE/durability	Rate	Favors vaccine
Son, K. Academic 2022 [40]	HIC (South Korea) Adults	Multiple: AstraZeneca, JNJ, Moderna, Pfizer Primary No vaccine	Through June 2022	Multiple estimates COVID‐19 infections and severe outcomes Not reported	Severe outcomes Clinical trials	Adverse Events (AE) and Severe AEs Clinical trials	Complex model Multiple criteria decision analysis (deterministic)	Varied benefit and risk weights	Weighted preference score	Favors vaccine
Stein, M. Academic/Health Authority 2022 [41]	HIC (Israel) Children	Pfizer Primary No vaccine	Delta	87.9%–100% COVID‐19 cases Not reported	Hospitalizations, ICU admissions, Long COVID, and MIS‐C Publications	Myocarditis Publications	Simple calculation (deterministic)	N/A	Rate	Favors vaccine
Tran Kiem, C. Academic/Health Authority 2021 [42]	HIC (EU/France) Adults	AstraZeneca Primary No Vaxzevria distribution	May–September 2021	80% and 90% COVID‐19 infection and severe outcomes Not reported	ICU admissions and deaths National disease surveillance	TTS ICU and deaths Regional pharmacovigilance	Complex model Dynamic (simulation, probabilistic)	Age Transmission	Count	Favors vaccine depending on age
Wallace, M. ACIP Health Authority 2021 [43]	HIC (US) Children—Adults	mRNA Primary No vaccine	Through February 2022	87%–97% COVID‐19 hospitalization Not reported	Hhospitalizations National disease surveillance	Myocarditis VAERS	Simple calculation (deterministic) and Complex model Dynamic (simulation)	Age and sex	Rate	Favors vaccine
Wallace, M. MMWR Health Authority 2022 [44]	HIC (US) Adults	Moderna Primary No vaccine	May 2021	57.4%, 92.7%, 95.9%, 100% COVID‐19 infection, cases, hospitalization, deaths Not reported	Asymptomatic or symptomatic infections, hospitalizations and deaths Publications	Myocarditis Clinical trials, VAERS, and Vaccine Safety Datalink	Simple calculation—modified GRADE (deterministic)	N/A	Modified GRADE	Favors vaccine
Winton Centre Cambridge Other 2021 [45]	HIC (UK) Adults	AstraZeneca Primary No vaccine	Through April 2021	Not reported COVID‐19 ICU admissions 4 m	Cases, hospitalizations, and ICU admissions National disease surveillance	TTS National pharmacovigilance	Simple calculation (deterministic)	Age Transmission	Rate	No interpretation provided in report
Woodworth, KR. Health Authority 2021 [46]	HIC (US) Children	Pfizer Primary No vaccine	Through October 2021	90% COVID‐19 cases Not reported	Symptomatic infections National disease surveillance	Systemic Adverse Events (AE) and Severe AEs Clinical trials	Simple calculation—modified GRADE (deterministic)	N/A	Modified GRADE	Favors vaccine
Yogurtcu, ON. Health Authority 2023 [47]	HIC (US) Adults	Moderna Primary No vaccine	December 2021 Separately for Delta and Omicron	72% COVID‐19 hospitalization or death 5 m Other scenarios considered	Cases, hospitalizations, ICU admissions, and deaths National disease surveillance	Myocarditis/pericarditis cases, hospitalizations, ICU, and deaths FDA Biologic Effectiveness and Safety System and Vaccine Safety Datalink	Simple calculation (deterministic)	Age and sex N/A VE/durability	Rate	Favors vaccine

Abbreviations: ACIP, US Advisory Committee on Immunization Practices; AstraZeneca, AstraZeneca ChAdOx1‐S; EU, European Union; FDA, US Food and Drug Administration; GRADE, Grading of Recommendations, Assessment, Development, and Evaluations; HIC, High‐Income Country; ICU, Intensive Care Unit; Janssen, Janssen AD26.CoV2.S; MAH, Market Authorization Holderl; MIC, Middle‐Income Country; MIC‐C, Multisystem inflammatory syndrome in children; Moderna, Moderna CX‐024414; mRNA, messenger ribonucleic acid; N/A, Not applicable; Pfizer, Pfizer‐Biotech BNT162b2; TTS, Thrombosis with Thrombocytopenia Syndrome; UK, United Kingdom; US, United States; VAERS, Vaccine Adverse Event Reporting System; VE, Vaccine effectiveness.

High‐income countries were the setting for the majority of qBRAs (*n* = 34), including the United States (*n* = 14), the EU (*n* = 7), Australia (*n* = 6), the United Kingdom/England (*n* = 4), Israel (*n* = 2), and South Korea (*n* = 1). One qBRA was conducted in 13 middle‐income Eastern European countries. The remaining two vaccine qBRAs were modeling exercises with no specific location. Most qBRAs focused on either adults (*n* = 18) or children (*n* = 7) for the target population. The other qBRAs (*n* = 12) targeted all ages or did not specify a target population.

The most common COVID‐19 vaccines assessed were mRNA vaccines either together (*n* = 9) or separately (Pfizer‐Biotech BNT162b2: *n* = 8, Moderna CX‐024414: *n* = 2). AstraZeneca ChAdOx1‐S (*n* = 9) and Janssen AD26.CoV2.S (*n* = 4) were assessed independently or simultaneously with at least one mRNA vaccine (*n* = 5). Most qBRAs focused on primary regimens (*n* = 36); however, some reports (*n* = 4) assessed booster doses alone or along with primary vaccination series. Unvaccinated individuals were the most common comparison group (*n* = 33).

Details on the data sources used to develop qBRAs were not always provided or complete. Vaccine efficacy/effectiveness measures were primarily based on published results of clinical trials or real‐world evidence studies, whereas most benefit and risk measures relied on Health Authority data. The benefit measures of averted COVID‐19 outcomes were most often derived from publicly available Health Authority surveillance system dashboards (e.g., US Centers for Disease Control and Prevention [48] and the European Centre for Disease Prevention and Control [49]). Risk measures were often extracted from public spontaneous pharmacovigilance databases including WHO Vigibase [50], US Vaccine Adverse Event Reporting System (VAERS) [51], or EudraVigilance [49] operated by the European Medicines Agency.

The most analyzed benefits of COVID‐19 vaccines were averted COVID‐19‐related events including death (*n* = 26), hospitalization (*n* = 24), ICU admission (*n* = 19), and infection (*n* = 14). Other potential benefits assessed included averted COVID‐19‐related blood clots, multisystem inflammatory syndrome in children, and pregnancy outcomes such as preterm birth and fetal demise. Vaccine‐related risks assessed included myocarditis and/or pericarditis (*n* = 19), thrombosis with thrombocytopenia syndrome (TTS) (*n* = 15), and Guillain‐Barre syndrome (*n* = 3). Other risks considered included anaphylaxis and coagulation disorders such as thromboembolic events, disseminated intravascular coagulation, cerebral venous thrombosis, and blood clots.

The qBRAs applied various analytic approaches; however, most assessments used simple calculations (*n* = 25), defined as descriptive statistics often conducted on aggregated data of static, closed populations. Most qBRAs performed with simple calculations were stratified by age and sex (*n* = 12), age (*n* = 4), or sex (*n* = 2). Five of the nine qBRAs using simple calculations that did not stratify by age were conducted among age‐specific populations of children (5–11 or 12–17 years old). Many qBRAs performed with simple calculations also accounted for sources of variability including different viral transmission scenarios (*n* = 7) and/or vaccine effectiveness (duration or waning) (*n* = 9).

The qBRA that employed more complex modeling methods (*n* = 15) used compartmental models, Monte Carlo simulations, Bayesian network models, and statistical analysis. While most reports (*n* = 26) summarized benefit and risk measures as rates per vaccine recipients or vaccine doses administered, some presented different measures including benefit–risk ratios (*n* = 4), modified Grading of Recommendations, Assessment, Development, and Evaluations (GRADE) or multiple‐criteria decision analysis (MCDA) scores (*n* = 3), numbers needed to treat/harm (*n* = 2), counts (*n* = 1), or surface of equipoise (*n* = 1).

Interpretation of the benefit–risk balance differed across qBRAs, mainly depending on the age of the vaccinees. Of the 37 selected qBRAs, 6 did not provide any interpretation of the benefit–risk balance of vaccination. Of the 31 qBRAs that interpreted the balance of risks and benefits, most (*n* = 21) concluded that the benefits of vaccination outweighed the risks. Four reports interpreted the qBRA results differently by age with all stating that the benefits did not outweigh the risks for younger populations while supporting vaccination in older age groups. Another two reports that were specifically conducted on children or young adults concluded that the risks of vaccination outweighed the benefits. One report favored vaccination for high‐risk individuals only. Three of the reports favored one vaccine over another.

## 4 Discussion

To our knowledge, this is the first systematic review summarizing quantitative approaches and methods that have been used to assess the benefit–risk profile of COVID‐19 vaccines. The urgency of COVID‐19 vaccine implementation required immediate and ongoing safety monitoring especially when vaccination was primarily implemented through mass vaccination campaigns. Many qBRAs were rapidly developed as part of early COVID‐19 vaccine monitoring, which likely drove the choice of input data (clinical trials reports, pharmacovigilance repositories, and COVID‐19 surveillance) and the use of rather simple analytical approaches. More complex models, such as compartmental or statistical models, take time to develop, test, and implement. Future COVID‐19 vaccine qBRAs could use more advanced methods to address additional degrees of complexity, such as further evolution of the SARS‐COV‐2 virus into new variants and related symptom severity, changes in vaccination recommendations, new or updated vaccines and their effectiveness against different COVID‐19 severity levels and SARS‐CoV‐2 infections, reduced surveillance for COVID‐19, new understanding of long COVID or post‐COVID conditions, potential impact of herd immunity and of natural immunity from previous SARS‐CoV‐2 infections, and findings from observational postauthorization vaccine safety and effectiveness studies. Future methods could include meta‐analytical approaches to integrate data, probabilistic modeling that accounts for uncertainty in the estimation process, and viral transmission dynamics simulations.

To some extent, many of the qBRAs were able to account for complexity by applying stratification, scenarios with varying parameters, and/or sensitivity analysis for key elements including age‐specific effects, waning of vaccine effectiveness, and changes in viral transmission rates. Stratifications by age and sex proved to be important due to higher and more severe COVID‐19 burden on the elderly [8, 48] and increased vaccine‐related risks of myocarditis and pericarditis in adolescent and young adult males [52, 53] and TTS mainly in adults under 50 years old [33, 54].

Identified qBRAs primarily focused on severe outcomes including hospitalizations, ICU admissions, and deaths, for benefits as averted COVID‐19‐related events and as risks associated with COVID‐19 vaccination. These severe outcomes not only demonstrate the impact on public health and clinical infrastructure but were also reliable and readily available measures during this pandemic, which is not always the case when conducting vaccine qBRA. Some qBRAs assessed milder outcomes such as COVID‐19 infections, which often lack the determination as symptomatic or asymptomatic and suffer from inconsistent or unreliable tracking over time and across jurisdictions. None of the qBRAs that relied on COVID‐19 infection surveillance data accounted for potential under‐ or overreporting of mild COVID‐19 infection, which could have been addressed in sensitivity analyses.

With the vast majority of qBRAs targeting high‐income countries, there is limited understanding of the balance of benefits and risk in low‐ and middle‐income countries (LMICs). Data availability and quality influence the feasibility of conducting qBRA in LMIC, but COVID‐19 vaccine introduction into high‐income countries first and lower uptake in LMIC [55] and restriction to reports available in English also likely influence this bias. Cross‐vaccine company initiatives were started during COVID‐19 to further consider ways to explore means to gather reliable and robust information in LMICs [56].

The consistency of the details included in qBRAs was minimal with different parameters used to assess benefits and risks, limited description of methods, and inconsistently presented results. Comparison across qBRAs can be challenging due to this heterogeneity and could affect the implementation of recommendations. These inconsistencies suggest that these reports were not developed with consideration of reporting guidelines such as the FDA Guidance Benefit–Risk Assessment for New Drug and Biological Products [57], the BRIVAC checklist [58] or the recently published Benefit–Risk Assessment of VAccines by TechnolOgy (BRAVATO) Standardized Module [7], which includes standardized fields that can be completed for the planning, conduct, and evaluation of vaccine benefit–risk assessments.

Despite efforts to assure the quality of this COVID‐19 vaccine qBRA review, this work has limitations. Some qBRA reports were likely missed even though extensive efforts to systematically identify qBRAs were employed. With nearly 1500 titles and abstracts reviewed, those that did not include the words “benefit” and “risk” could have been eliminated. The BRIVAC checklist specifically recommends titles include “quantitative benefit–risk model” [58]. Our review was able to identify most key elements in the selected qBRAs, but our interpretation of the provided detail could fail to accurately represent the truth either due to our misinterpretation or lack of sufficient information in the report.

Given that the reviewed qBRAs covered the early phases of COVID‐19 vaccine introduction, it is fortunate that most concluded that the benefits of COVID‐19 vaccines outweighed risks at least in some segments of the population. Future qBRA for vaccines targeting new SARS‐CoV‐2 variants or other pathogens (e.g., respiratory syncytial virus or influenza) will likely not be able to leverage such robust and timely data as was available during the early COVID‐19 pandemic. The COVID‐19 pandemic highlighted the importance of robust, timely, and readily available public health surveillance data, which can be used for many activities including qBRA. Efforts to modernize public health data [59] and those to increase trust and use of real‐world evidence [60, 61] are important for future vaccine qBRAs.

## 5 Conclusion

The high number of qBRAs identified in this review demonstrates the need to rapidly assess the benefit–risk profile of newly introduced vaccines for informed and timely public health decision‐making. This comprehensive review and critical assessment of COVID‐19 qBRAs together with available guidance should be used to support the robust and transparent development of future vaccine qBRA.

### 5.1 Plain Language Summary

With the introduction of COVID‐19 vaccines, there has been a proliferation of initiatives aiming at comparing benefits and risks of vaccination in a quantitative manner. The aim of this article was to systematically identify, review, and critically assess published COVID‐19 vaccine quantitative benefit–risk assessments. Through systematic review of the literature, screening of selected Health Authority websites, and a grey literature search, we identified 37 studies reporting on quantitative benefit–risk assessments. The latter were conducted on two mRNA and two adenoviral vector COVID‐19 vaccines. Only one study represented low‐ and middle‐income countries. Although many qBRAs used simple methodological approaches (*n* = 25), more complex estimation models were presented in 15 reports. Simple approaches were able to employ stratification by age and/or sex to highlight safety issues affecting specific demographic groups and scenarios to account for changes in viral transmission and vaccine effectiveness over time. Details regarding data sources and analytic methods were missing or limited in some reports. This comprehensive description and critical assessment of COVID‐19 vaccine quantitative benefit–risk assessments together with available guidance can be used to support the development of robust and transparent future similar assessment.

## Ethics Statement

The authors have nothing to report.

## Conflicts of Interest

N.P., K.H., C.W., L.W., and B.L. are employees of Johsnon & Johnson Innovative Medicine and may hold stocks in the Company. ECN was an employee of Johsnon & Johnson Innovative Medicine. She is now an employee of Moderna Therapeutics and may hold stocks in these Companies. RV was a trainee at Johsnon & Johnson Innovative Medicine, for which she received some financial support from the Company.

## Supporting information


**Data S1.** Search strategy per database.
