# Supplementary material for: Quantitative Benefit–Risk Assessment of Vaccination Against COVID‐19: A Systematic Review
Source: Pharmacoepidemiol Drug Saf. 2025 Jan 29;34(2):e70099. doi: 10.1002/pds.70099 (PMC11779546; doi:10.1002/pds.70099)
Supplement: Supplementary file 1 — Data S1. Search strategy per database. [file PDS-34-e70099-s001.docx]

Supporting Information. Search strategy per database.

| Database | Search Strategy |
| --- | --- |
| medRxiv | abstract or title "COVID, benefit, risk, vaccine" (match all words) |
|  | abstract or title "covid-19, benefit, risk, vaccine" (match all words) |
|  | abstract or title "covid, benefit, risk, vaccination" (match all words) |
|  | abstract or title "covid, benefits, risks, vaccine" (match all words) |
|  | abstract or title "covid, benefits, risks, vaccination" (match all words) |
|  | abstract or title "corona, benefit, risk, vaccine" (match all words) |
|  | abstract or title "corona, benefit, risk, vaccination" (match all words) |
|  | abstract or title "SARS-CoV-2, benefit, risk, vaccine" (match all words) |
|  | abstract or title "SARS CoV 2, benefit, risk, vaccine" (match all words) |
|  | abstract or title "SARS CoV 2, benefit, risk, vaccination" (match all words) |
|  | abstract or title "SARS CoV 2, benefits, risks, vaccination" (match all words) |
|  | abstract or title "SARS CoV 2, benefits, risks, vaccine" (match all words) |
| PubMed | **#1 COVID-19**  (("COVID-19"[Mesh] OR "SARS-CoV-2"[Mesh] OR "COVID-19 Vaccines"[Mesh] OR "COVID-19 Serological Testing"[Mesh] OR "COVID-19 Nucleic Acid Testing"[Mesh] OR "SARS-CoV-2 variants" [Supplementary Concept] OR "COVID-19 drug treatment" [Supplementary Concept] OR "COVID-19 serotherapy" [Supplementary Concept] OR "2019-nCoV" OR "2019nCoV" OR "cov 2" OR "Covid-19" OR "sars coronavirus 2" OR "sars cov 2" OR "SARS-CoV-2" OR "severe acute respiratory syndrome coronavirus 2" OR “coronavirus 2” OR “COVID 19” OR “COVID-19” OR “2019 ncov” OR “2019nCoV” OR “corona virus disease 2019” OR “cov2” OR “COVID-19” OR “COVID19” OR  “nCov 2019” OR “nCoV” OR “new corona virus” OR “new coronaviruses” OR “novel corona virus” OR “novel coronaviruses” OR “SARS Coronavirus 2” OR “SARS2” OR “SARS-COV-2” OR “Severe Acute Respiratory Syndrome Coronavirus 2”) AND (2019/12/1:3000/12/31[PDAT])) |
|  | **#2 benefit-risk**  (("benefit" [TIAB] AND "risk" [TIAB]) OR ("benefits" [TIAB] AND "risks" [TIAB]) OR ("benefits" [TIAB] AND "risk" [TIAB]) OR ("benefit" [TIAB] AND "risks" [TIAB]) OR ("benefit-risk" [TIAB]) OR (“risk-benefit" [TIAB])) |
|  | **#3 Vaccine**  ((“vaccines” [Mesh]) OR (“vaccine” [TIAB] OR “vaccines” [TIAB])) |
|  | **#4 Combining and refining**  (#1 AND #2 AND #3) Filters: Abstract, Classical Article, Clinical Study, Dataset, Meta-analysis, Observational Study, Review, Systematic Review |
| ScienceDirect | (immunisation OR immunization OR vaccine OR vaccination) AND benefit AND risk AND (COVID OR SARS-CoV-2 OR corona) |
| Health / Regulatory Authority website search | (“Covid-19 vaccine” and "benefit - risk") and  (“Australia” and "TGA”) or  (“European Union” and "EMA”) or  (“Japan” and "PMDA”) or  (“United Kingdom” and "MHRA”) or  (“United States” and (“FDA” or “CDC”))  (“World Health Organization” or “WHO”)) |
| General internet search | “Covid-19 vaccine” and "benefit – risk assessment" and “quantitative” |
